# Rerouting Geriatric Medicine by Complementing Static Frailty Measures With Dynamic Resilience Indicators of Recovery Potential

**DOI:** 10.3389/fphys.2019.00723

**Published:** 2019-06-19

**Authors:** Marcel G. M. Olde Rikkert, René J. F. Melis

**Affiliations:** Department of Geriatrics, Radboudumc Alzheimer Center, Radboud University Medical Center, Nijmegen, Netherlands

**Keywords:** frailty, resilience, forecasting, older adults, tipping points

## Abstract

Medicine is still very inadequate in forecasting recovery of tipping points in health and disease, especially in older adults. However, increasingly, diseases and invasive treatments unexpectedly push older patients with low resilience over their tipping points (TPs). These TPs are the points in human physiology that separate more healthy conditions from disease conditions or malfunctioning of the older human’s subsystems or organs, such as delirium, syncope and falls in old age, which threaten the functioning of the older person as a whole. Either the person may recover from the perturbation induced by such a subsystem TP and the balance of the whole system is restored, or the TP may set in motion a cascade of events driving the system down to a state of more decline, ultimately leading to death. A main unanswered question here is how to predict whether these older persons will recover or not. To improve this TP-recovery-forecasting, intriguing findings on measures of resilience found in other complex biological systems may be translated to humans. New dynamic resilience biomarkers for resilience can enrich clinical prediction for pathophysiological recovery and could test interventions for their effectiveness in improving resilience. Therefore, we hypothesize that dynamic, stimulus-response measures of recovery rate over time, observed after having received a minor stressor in a healthy condition, can be used to quantify recovery potential following subsystem TPs in disease and following invasive treatments in humans and thus the person’s resilience. Current static frailty prognostics can predict risks for death, institutionalization, delirium, falls, and other TP transitions, but it has not been proven that they can predict recovery. Our hypothesis on dynamic indicators of recovery is logical and timely, as it can now be studied with sensor technology to create a fundamentally different approach of variables that may be validated to forecast recovery potential. By generating dynamic measures of systemic resilience over various organ systems, we may subsequently model resilience generically across many chronic diseases, affecting different organ systems. Next, quantifying systemic resilience may reroute scientific and clinical pathways by predicting and preventing irreversible tipping points and by improving recovery by older adults.

## Introduction

Due to increased life expectancy, the number of persons aged 80 and over in Europe is predicted to rise with 250% within this century^1^. One of the hallmarks of this aging trend is the importantly increasing heterogeneity of the population, as aging increases between person variability of most biological and psychological parameters (Olde Rikkert et al., 1997). However, apart from age and demographic variables, we often lack accurate information on the dispersion measures of physiological processes between older adults (Olde Rikkert et al., 1997). In this heterogeneity, “resilience” – in essence, the individual’s recovery potential and restore to its current state – may complement the term “frailty” and improve characterization of the differences in recovery potential between aging individuals. The linked concept of frailty is most often defined as a condition of being more susceptible to a diseased state due to decreased adaptability to stressors (Clegg et al., 2013). Current static frailty prognostics can predict risks for delirium, falls, and other frequently occurring subsystem tipping point (TP) transitions in older adults and ultimately also death. TPs are the points in time that separate a more healthy condition from an acute, but in principle reversible disease condition and malfunction of the human’s subsystems or organ dysfunction (e.g., delirium, a fall or a depressed state). Therefore, TPs are critical challenges to the older human’s equilibrium state (Scheffer et al., 2001).

Improved understanding and forecasting of resilience as recovery potential from these TPs probably requires dynamic measures, reflecting the person’s change over time following a stressor. New dynamic resilience biomarkers (e.g., slowing down in blood pressure, temperature or glucose response after a standardized physiologic stressor) for resilience can enrich clinical prediction for pathophysiological recovery and could test interventions for their effectiveness in improving resilience. Such dynamic measures, i.e., measures with a time scale, should logically be the first to be tested as predictors of recovery, which is also a process over time, to which dynamic resilience measures conceptually can be most closely defined (Gijzel et al., 2017). Dynamic resilience can be quantified by analyzing time series in hemodynamics using indicators such as blood pressure, heart rate, and related variables, but also cerebrovascular responses can be simply and reliably studied over time following clinically relevant micro-stressors, such as changing posture or performing hyperventilation.

Ultimately, clinical care might benefit greatly from improved forecasting of resilience regarding the frequently occurring health crises, such as falls and delirium, as it may improve personalization of recovery assisting interventions for older adults (Olde Rikkert et al., 2016). In addition, such forecasting may improve the selection of older patients for obtrusive surgical and oncologic interventions based on sufficiently high chances for recovery, as promoted, for example, for vascular surgery in the oldest old (Ali et al., 2018). Such medical interventions may be reconsidered or even withheld from older adults with insufficient resilience, because such patients face the risk of being pushed over their TPs into an acute and irreversible decline of their health, specifically by delirium, falls, depression, and loss of mobility. To raise a new perspective for quality of care improvement in medicine for the aged, this paper describes the concept of generic resilience for recovery of TPs and presents a new hypothesis of how this generic and subsystem resilience may be measured best.

## Resilience Concept

The concept of systemic resilience appears to be straightforward. However, it is in fact not obvious that a single overarching system property exists that determines the risk of passing through and recovering from TPs for the most important disease states in older adults, such as delirium, depression, falls, and acute loss of autonomy in activities of daily life. In principle, these acute severity states are reversible, but ultimately they may also lead to death from a cascade of complications.

Historically, resilience in humans was first defined as the systems’ ability to cope with stress and preserve functioning (McEwen, 2004). Since then, systemic resilience has been predominantly studied in the stress recovery system of the hypothalamic-pituitary-adrenal (HPA) axis. Later on, resilience was studied in depth in medicine in the domains of psychology and psychiatry, where it is defined as the capacity to recover following psychosocial stress (Lavretsky, 2014).

A growing series of recent biological empirical studies returned to the original concept of systemic resilience and showed that this may be quantified by several mathematical measures of slowing recovery of complex systems after perturbation by stressors, which both may be artificial (heat, chemicals) and natural (climate change or disease) (Scheffer et al., 2001, 2009, 2012). The validity of such measures of slowing down of recovery already is confirmed by controlled laboratory experiments, initially with cyanobacteria and algae (Carpenter et al., 2011; Veraart et al., 2011).

Added to this, powerful recent evidence for the existence of systemic resilience in living systems comes from work on the nematode worm *Caenorhabditis elegans*, which probably is the best studied animal model in aging research. For example, 100,000 of these worms were exposed to stimuli such as heat, chemicals, food, and mutagens to challenge resilience. The study showed that survival curves were of similar shape, regardless of the stressor (Stroustrup et al., 2016). This strongly suggests the existence of a systemic physiological property – systemic resilience – which causes similar defense reactions to different stressors. These preclinical nematode experiments support the hypothesis that various kinds of (micro-)stressors may be used to quantify systemic resilience in more complex biological systems. To gain more understanding of the underlying physiology of systemic resilience in man, it is best to properly define resilience, as the whole persons’ capacity to recover from challenges, and regain the previous levels of physical and mental condition, and of autonomy in activities of daily living, ultimately determining the chances of survival. Complementary, subsystem resilience refers to the recovery potential of physiological subsystems such as postural balance, blood pressure, cerebral perfusion or mood. Resilience as physiological measure related to the dynamical systems theory may be used to generate useful mathematical models that can accurately describe behavior of complex dynamical systems in biology, such as frequently occurring TP transitions in nature (Scheffer et al., 2001). However, so far, this TP-related theory has not yet been used to describe age-related diseases, nor recovery potential in disease trajectories and physiology of aging. By adopting this system dynamics perspective of resilience, it may be more usefully applied in man, probably most valuable in older adults as they frequently pass subsystem TPs related to acute severity changes of chronic diseases (e.g., acute renal failure on top of chronic renal failure, or delirium in case of dementia). These TPs seriously threaten the integrity of overall equilibrium state, and thus survival, if not recovered from.

Similar to other complex systems, humans also pass through critical shifts between alternative states (Dakos et al., 2012). In many human diseases, self-propagating positive feedback mechanisms can cause unstoppable TP transitions. For example, a nasty remark to a person in a labile subnormal mood may exacerbate social isolation, resulting in fewer positive interactions and more negative feelings, which finally propagate into a severely depressed state (van de Leemput et al., 2014). From this perspective, a number of striking similarities can be found between different warning signals for impending acute transitions during chronic episodic disorders (Olde Rikkert et al., 2016). For example, elongation of the recovery period of an acute heart failure episode acts as risk marker for quick relapse of heart failure (Olde Rikkert et al., 2016), longer recovery time of repolarization in cardiac muscle cells (longer QTS interval) increases the risk of ventricular fibrillation (Lahti et al., 2014) and longer recovery times in epilepsy (Kramer et al., 2012), and migraine (Scheffer et al., 2013) predict subsequent seizures and headache attacks, respectively (Table 1). This evidence base can be used to develop a groundbreaking new hypothesis to quantify systemic resilience in human physiology.

**Table 1:** Preliminary evidence for recovery time as an indicator of resilience and prognosis for recovery after passing TPs in the average course of a range of chronic diseases.

Discipline	Disease	Recovery time	Disease state predicted by longer recovery time
Cardiology	Arrhythmia	QTc elongation time	Torsade de Pointe arrhythmia
G-enterology	Colitis	Clearing time Clostridium Dif.	Clostridium Dif. overgrowth
Geriatrics	Falls	Centre of mass recovery time	Falls, loss of balance
Hematology	Acute leukemia	Lymphocyte recovery time	Relapse of disease
Immunology	Breast cancer	Lymphocyte recovery time	Relapse of disease
Neurology	Epilepsy	Seizure recovery time	Epileptic state
Oncology	Neck cancer	Lesion regression time	Relapse state
Psychiatry	Depression	Positive mood recovery time	Depressed state
Public health	Smoking	Craving decay over time	Relapse of smoking
Pulmonology	Tube-ventilation	Ventilation recovery time	Ventilation weaning failure

For evidence base, see Stratton et al., 1982; Fish et al., 2004; Seymour et al., 2008; Kubicki et al., 2012; Lahti et al., 2014; van de Leemput et al., 2014; Matoba et al., 2015.

## Frailty

According to the official EU statistics, 25.8 million Europeans were age 80 and older in 2015. Of 67.9 million Europeans aged 65–79 years, an estimated 7.5 million (5%) may be frail (Clegg et al., 2013). In the group older than 80 years, 16% is estimated to be frail, and in total, this group is growing rapidly. Frailty is mostly defined epidemiologically as a health state with high risk for negative outcomes following minor illness or trauma (Clegg et al., 2013; Rockwood and Mitnitski, 2015). Described in the context of this paper, the major problem in these frail adults is that they are highly prone to multiple TP incidents. The incidences of the most important TP incidents within 3 months after hospital discharge are high, but also highly variable: delirium: 20–29%, falls: 11–20%, and acute functional decline: 11–60% (Bakker et al., 2014; Inouye et al., 2014; Bakker and Olde Rikkert, 2015). In 25–40% of the subjects, this resulted in sudden and substantial loss of well-being (Steptoe et al., 2015). These high rates indicate that acute changes in health are a major burden for older adults, their families, and society. Improved prediction models are therefore urgently required. Currently, medical practitioners are unable to identify which older patients are close to their TPs, nor whether they have the power to recover from passing these TPs, and benefit from treatment (van Kempen et al., 2015). There are several reasons why identifying resilience in individual patients requires innovation of the current frailty measures, which predominantly have epidemiological aims (Rockwood and Mitnitski, 2015). The frailty measures are mostly based on simply summing up the patients’ static deficits, which can only predict major negative outcomes such as mortality and hospitalizations, and predict only at the group level (between patients), not for individual patients (within patients) (Bakker and Olde Rikkert, 2015; Gill et al., 2015). The more than 50 measures of frailty that have been developed in the last two decades are all based on a static risk assessment, calculated at a single time point (Hamaker et al., 2012; Clegg et al., 2013; Hubbard and Jatoi, 2015; Hoogendijk, 2016). None of these measures monitors or predicts within patients’ (change in) recovery over time after passing from a healthy to a disease state, i.e., after passing a TP. Moreover, frailty indicators were not validated to predict recovery (Bakker and Olde Rikkert, 2015). Finally, innovation is needed because not only deficits are relevant for recovery potential but also positive capabilities such as coping ability, fitness level, and having goals in life (Crimmins, 2005; Warnier et al., 2016). Mechanistically, it makes sense that recovery potential is not simply the reverse of physical frailty.

Having to face an increasingly aged population, recognizing frailty has already resulted in major advances in geriatric medicine. Currently, frailty is most precisely measured by the frailty index (FI) based on accumulation of health deficits (Rockwood and Mitnitski, 2015; van Kempen et al., 2015). This FI quantitatively assesses an individual’s clinical health state, including symptoms, clinical signs, laboratory abnormalities, diseases, and disabilities. However, since frailty criteria are mostly based on the consequences of functional losses, an FI fails to identify individuals at risk before changes are apparent during clinical examination. Recently, Mitnitski et al. called for the development of an earlier measure of frailty and therefore proposed a biomarker-based FI (Mitnitski et al., 2015). Although this advanced FI may indeed contribute to early recognition of frailty, it does not help to enhance understanding of TPs recovery potential in humans. Moreover, the currently used FI’s conceptually do not capture the variable and often non-linear changes in vulnerability over time in a single patient (Varadhan et al., 2008; Clegg et al., 2013). This is because, so far, it is impossible to measure frailty in a dynamic way.

In agreement with this, Fried’s research group suggested that frail and non-frail adults may be differentiated much better by assessing the dynamics of their physiological systems’ response to stimuli, instead of the current frailty assessment at only a single time point (Varadhan et al., 2008). However, the theoretical, practical, and analytical difficulties associated with such a dynamic approach to frailty so far have limited scientific progress in this direction. This is underlined by a systematic review of frailty studies, in which not a single example of such a dynamic approach to frailty was found (Clegg et al., 2013; Hubbard and Jatoi, 2015). In the following subsection, the concept of subsystem TPs is linked to frailty and resilience and it is shown how this enables a common design for testing resilience in the equilibrium states of, for example, blood pressure, heart rate, and mood.

## Tipping Points

The existence of TPs is easily illustrated by the fainting patient in whom blood pressure and cerebral perfusion has declined below the lowest limit of cerebrovascular autoregulation. Fainting occurs after passing a reversible TP. It may seem counter-intuitive that human beings in fainting would show the same resilience properties as simpler organisms such as blue-green algae, in which the first evidence for TPs was reported (Scheffer et al., 2001, 2012). However, it is becoming increasingly evident that many complex systems have critical thresholds, or TPs, during which the system shifts abruptly from one state to another. As they approach the TP, these shifts become more self-propelling. In medicine, examples of such systemic reversible states include delirium episodes, heart failure crises, recurrent falls, migraine attacks, and epileptic seizures. Intriguingly, similar TPs have been demonstrated in global finance by the recent systemic bank and market crashes (Battiston et al., 2016), in historical ice-age climate changes (Scheffer et al., 2012); and in sudden breakdowns in a range of ecological systems, for example, in the alarmingly fast loss of vitality of the Great Barrier Reef (van de Leemput et al., 2015). The common denominator of complex systems in which these TPs of acute and theoretically reversible change are observed is that they all rely on one or more positive feedback mechanisms. These can accelerate change and propel the system over the tipping into another less preferable, but more or less stable, diseased state.

**Figure 1:**
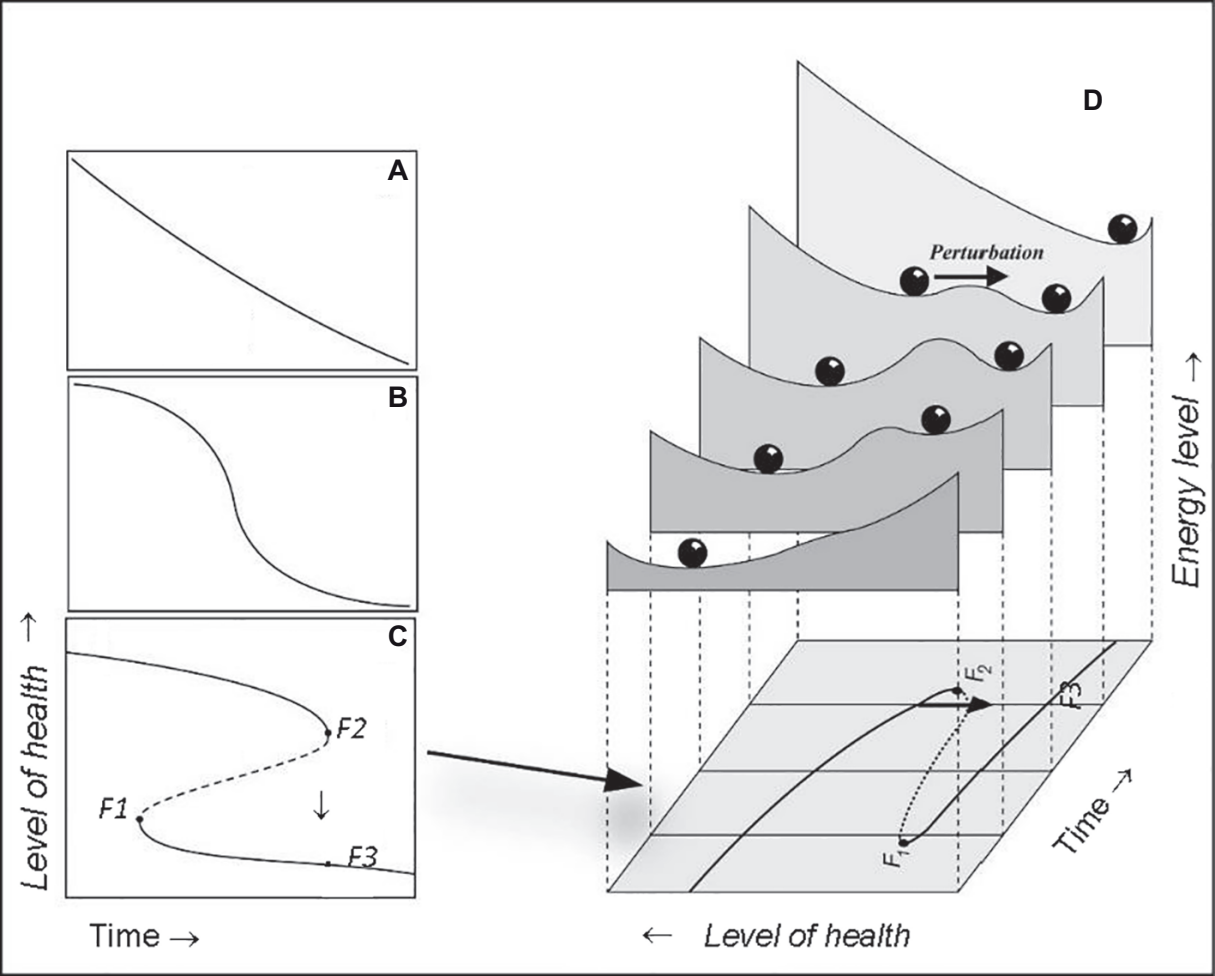
Catastrophic bifurcations in human physiology are illustrated by the well-known fold catastrophe (left hand panels). Although some systems respond smoothly **(A)** over time, change can also be relatively sharp over time in the equilibrium curve, which reflects the response of the organ or individual around some threshold condition **(B)**. The situation in which critical transitions can occur arises if this equilibrium curve is “folded” **(C)**. Then three equilibria can exist for a given condition. If the system is very close to a bifurcation point (i.e., *F2*) a tiny change in the condition, may cause a large shift in health state to the lower branch [*F3* in **(C)**]. Close to such a bifurcation a perturbation [arrow in right hand graph **(D)**] can easily push the system across the boundary between the attraction basins, as illustrated by the stability landscapes in the right hand graph. Subjects may then suddenly shift from one to another health state and energy level. The likelihood of such a sudden change over time differs with the system’s resilience: least resilient close to the tipping point (landscape slide with arrow) more resilience moving away from this point (*F2*), backward and forward (so non-linear). When the route would be started from the right hand side [at *F3* in **(C,D)**] backwards, the way up would lead to point *F1*, and therefore would be different from the way down (*via F2*). This shows that bifurcation points are passed differently coming from different directions. In human physiology this is resembled by the fact that the recovery route differs from the route of acutely falling ill.

Within dynamic systems theory, the mathematical catastrophe model can help to illustrate how changes in an adults’ systemic resilience act as risk markers of increased likelihood of passing TPs. In this model, TPs are known as catastrophic bifurcations (Veraart et al., 2011). Although there are several types of catastrophic bifurcations, the idea is most easily illustrated by looking at the well-known “catastrophe fold” model (Figure 1). To understand this model, note that the equilibrium state of an older patient can respond in different ways to stressors such as, for example, pre-surgical anesthesia, surgery or chemotherapy (Figures 1A–C). Although some older patients with sufficient resilience respond smoothly over time (Figure 1A), change can also be relatively sharp in patients with low resilience around some threshold condition (Figure 1B). The situation in which critical transitions occur, for example, toward a syncope state or a stroke, can be modeled by an equilibrium curve which is “folded” (Figure 1C). In short, there are three models of adaptation in health states over time of a person who is in equilibrium with a stressor. The model of acute change adaptation (Figure 1C) best illustrates older patients with insufficient resilience having to stand a new disease or an intervention. Their physiological systems are closest to the bifurcation point (i.e., *F2*). Consequently, a small change in their condition causes a large shift in their level of health to the lower branch of the model (from *F2* downward to the lower line at *F3* in the same time point, in Figure 1C).

Similarly, when a patient is close to such a TP bifurcation, a minor stressor can easily push the system across the boundary between the basins of attraction, as illustrated by the stability landscapes in Figure 1D. These bifurcation points are the TPs where the self-propelling change can produce a large transition in response to a minor health stressor. In older adults, such a large transition may result in sudden syncope, atrial fibrillation or delirium. Although declining systemic resilience may seemingly have little effect on older patients, it turns out to result in a situation where even small stressors, no matter what sort, may push these patients over a TP, to show the lack of resilience of the older individual.

## Comparison of Resilience and Frailty

In aging research, resilience has been used in psychology and was related to positive factors determining recovery such as strength, immunity, coping behavior, optimism, and good cognition (Richardson, 2002; Ahern et al., 2006; Lavretsky, 2014). This fundamentally distinguishes resilience from frailty, which primarily was defined for physical changes, and was validated only for predicting negative outcomes such as death and hospitalization, and which only takes account of deficits (Clegg et al., 2013). This difference between frailty and resilience is reflected by the six currently available resilience scales, which all use different, positive psychological factors predicting recovery. However, similar to frailty scales, current resilience scales limit their measurements to a single point in time (see Table 2; Lavretsky, 2014).

**Table 2:** Comparison of the current state of measurements of frailty and resilience.

	Measures of resilience	Measures of frailty
**Static**: list of items questioned or observed	Predict positive outcomes (recovery). Currently, 6 psychological resilience scales are in use (e.g., Conner-Davidson resilience questionnaire)	Predict negative outcomes, e.g., mortality. More than 50 static frailty scales are currently available (e.g., Fraily index or Fraily phenotype)
**Dynamic**: monitoring stimulus-response over time	Dynamic measures of resilience are not yet available: the hypothesis to develop and validate these is proposed here (e.g., response of blood pressure on orthostatic response over time)	Dynamic measurements of frailty are not available

While normal aging results in only gradual loss of systemic resilience, this can happen more quickly and unpredictably if aging is accelerated by disease. If the risk for systemic failure is high, young resilient patients usually are transferred to the intensive care. Successful transfer of geriatric patients to an intensive care ward might be improved by the assessment of resilience beforehand, but this assessment is lacking in current practice. Not surprisingly, in medicine, the efforts to understand systemic resilience are most advanced in geriatrics and critical care medicine. In the face of a rapidly aging population and a broad range of public health challenges, there is a widespread and growing interest in assessing resilience and unraveling factors that contribute to it. For instance, geriatricians have shown that gait speed and hand grip strength are good predictors of expected lifespan (Studenski et al., 2011). Qualitative checklists on resilience also allowed a reasonable quantification of overall human health (Lavretsky, 2014). However, these are all indirect and static indicators of resilience.

**Figure 2:**
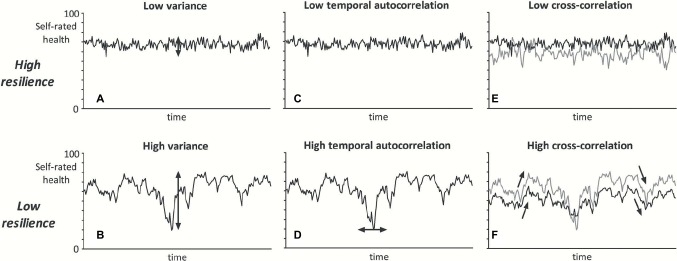
Graphical illustration of the dynamical resilience indicators. If a person has low systemic resilience, fluctuations of self-rated health over time are hypothesized to show higher variance **(A** vs. **B)**, higher temporal autocorrelation **(C** vs. **D)**, and higher cross-correlation between self-rated health domains **(E** vs. **F)**. In this example of fluctuations of self-rated health (data were randomly induced with a model), the black line may represent physical health and the gray line mental health (Gijzel et al., 2017).

An exciting and promising approach to quantify resilience is to measure the rates of recovery following a micro-stressor which causes a safe, micro-change in healthy state (Hadley et al., 2017). Originally, it was thought that this resilience could only be determined by invoking a shift in the biological system and measuring the magnitude of the stressor needed for that. However, it now appears that stochastic changes in the dynamics of biological systems may be used to quantify the chances for recovery after passing a TP, but without pushing the patient over the TP (Gijzel et al., 2017). The most straightforward dynamic indicator of resilience is slowing down of recovery (SDR). Simply put, SDR is the response to micro-changes close to a TP. Increased variance and higher autocorrelation in individual time-series of variables that reflect low system resilience have shown to be highly correlated with SDR (Figure 2) and may have similar predictive value (Veraart et al., 2011; Gijzel et al., 2017).

**Figure 3:**
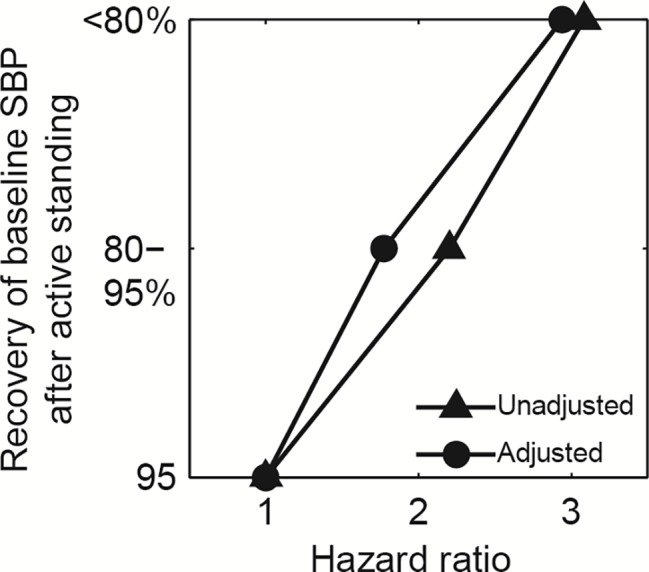
Slowing down in recovery patterns of systolic blood pressure (SBP) after active standing. Lines show Cox proportional hazards ratios for all-cause mortality, unadjusted and adjusted for age, gender, body mass index, co-morbidity, SBP, diastolic BP (DBP), heart rate, and drug use (*N* = 238). Mortality is lower in the group with 95% recovery to baseline SBP in less than 1 min after standing (*N* = 95), compared to the group with 80–95% recovery (*N* = 98), and the slow (<80%) recovery group (*N* = 45), (*p* < 0.05) (Lagro et al., 2012, 2014).

Recently, we showed first proof of concept for the value of SDR while testing the parameters of blood pressure regulation (Lagro et al., 2014). If a person suddenly stands up, orthostatic blood pressure drops due to gravity. Normally, the body quickly corrects this by vasoconstriction and increased cardiac output. This rapid regulation often fails in older adults, leading to a drop in cerebrovascular perfusion, sometimes even resulting in a syncope. Remarkably, a slow rate of blood pressure recovery upon standing up was a significant predictor of recurrent syncope and all-cause mortality (Figure 3; Lagro et al., 2012, 2014). Thus, SDR in blood pressure regulation was a preliminary sign of lower systemic resilience connected to overall death rate and could be accurately measured with hemodynamic parameters. Similarly, time series of self-reported mood revealed that SDR in expressed emotions signaled a lower chance of mood recovery (van de Leemput et al., 2014). It thus appears that human beings are no exception to what may be a general rule in all complex dynamical systems that SDR in physiological key parameters can be a generic indicator of low resilience.

## Mapping Disease Trajectories

In our aging trajectories, most persons will pass several subsystem TPs. After passing a first TP, we often lose physical and/or mental capacities, and we may or may not recover completely. Now imagine that we would be able to individually forecast resilience, as measure of proximity to these TPs and the chances to successfully recover from them. This would significantly improve our prospects of retaining good health during aging, and it would help us to make complex health-related decisions to find the best route through the different individual landscapes of aging and disease. However, so far the exciting option of a generic resilience mechanism and tipping point physiology has scarcely been studied, even in older adults (Hadley et al., 2017), despite their high risk of passing TPs and the importance to be able to quantify their resistance to perturbations and recovery potential.

## Hypothesis of Forecasting Tipping Point Recovery

Therefore, we plead to study a dynamical and systemic resilience concept. Altogether, this leads to three closely linked sub-hypotheses:

1. Patients with high systemic resilience have low susceptibility for micro-stressors, which predicts lower risk of passing a TP and entering a diseased state.
2. Indicators of resilience in specific parameters (e.g., blood pressure, heart rate, and mood) predict systemic resilience in older adults. This is predicted by dynamic systems theory as it shows that biological parameters become more closely linked as the entire system becomes less stable (Asada et al., 2016; Cohen, 2016).
3. Dynamic measures of resilience improve forecasting compared to prognostics that are based on static measures of symptoms and vital signs, measured at a single time point. This is particularly important in elderly people because the predictive value of widely used static physiological measures, based on vital signs such as blood pressure and respiration rate, declines with age (Hubbard and Jatoi, 2015).

In sum, the overarching hypothesis is that compared to currently used static predictors, systemic, time-dependent measures of resilience may improve forecasting of the proximity of TPs and the recovery from passing these TPs in older patients.

## Challenges

There are several reasons why such hypothesis driven TP forecasting in older adults is still a major challenge:

1. It is notoriously hard to predict the timing of TPs, as ongoing health changes may quickly change the resilience of older patients. Even a transient flu or 2 weeks bed rest may lower resilience for elective surgery. So far, we lacked the technology of quickly responsive, sensor techniques, linked to stimulus-response monitoring, specifically in heart rate, balance, attention, and mood. These are needed to reliably track resilience over time in the parameters most important for older adults.
2. During intensive medical interventions such as chemotherapy or oncologic surgery, static measures of frailty have shown only limited predictive value for complications, adverse events and mortality, and no predictive value for recovery potential and beneficial outcome in surgical or oncologic trajectories (Hamaker et al., 2012; Hubbard and Jatoi, 2015). Moreover, the seven clinically applicable frailty instruments have a predictive accuracy of only 60% compared to detailed frailty instruments used in research (Warnier et al., 2016).
3. Integrated care is assumed to improve the outcome of complex medical problems for older adults (Bakker and Olde Rikkert, 2015). However, trials on integrated care, targeted by frailty level, did not yet result in an improved outcome of fewer TPs. For example, eight large trials targeting at frailty, from the recent Dutch National Programme on Aging, did not result in improved outcome (Hoogendijk, 2016). To improve outcomes, resilience-directed targeting criteria, and thus dynamic targeting measures, may be indispensable.
4. Individual modeling and forecasting is notoriously difficult. Even in intensive care medicine, the massive datasets of routinely collected time series are not yet transformed to validated, individual forecasting models. However, there is some proof-of-concept for this individual modeling by constructing individual time series in Bayesian modeling to understand multimorbidity and disease trajectories in chronic obstructive pulmonary disease (Lappenschaar et al., 2013; Bueno et al., 2016). In this approach, graphical and quantified models of time series were combined, which enabled input from mathematical formulae on the one hand, and mechanistic reasoning based on medical expertise on the other hand. This combined approach may make the uncertainty level of population-based forecasting applicable in individualized predictions (van der Heijden et al., 2014).

## Perspective

There is a risk that the hypothesized resilience and TP paradigm ultimately may not be confirmed in the context of geriatric medicine. However, even so the time series that will be studied by testing this hypothesis may provide unique data of important TPs, which can open new research avenues and clinical applications, just as the first real-life videotapes of falls did in falls research and prevention (Robinovitch et al., 2013).

In case the proposed hypothesis would be confirmed, this would be a major breakthrough as this would lead to improved individualized forecasting tools, to be used in a wide range of applications. As such, dynamic resilience assessment for specific pathophysiological or treatment stressors could expand the first start of personalized medicine in older adults, which can now only be based on the unified frailty state added to the classical disease, history, and demographic data. Resilience, however, can and should be assessed specifically for different stressors and could lead to different conclusions for psychological, psychiatric, and surgical stressors.

This could be operationalized with different time series such as in hospital early warning scores, body worn accelerometers, cardiovascular and metabolic sensors (temperature, glucose responsiveness, and sodium), and combinations of them. Many of these are already collected, but should be validated still for specific (patho)physiologic recovery processes. Such validation work could ultimately result in a toolbox of (sub-)resilience measures based on time series analyses techniques, of which a range of medical disciplines could profit, as many clinicians encounter TP dynamics.

Moreover, improved forecasting techniques could easily become cost-effective for our aging societies. If improved TP navigation in older patients would result in 1–5% reduction of the yearly 10–20% acute hospitalizations of frail older persons – modestly extrapolated – it could reduce Europe’s health (Inouye et al., 2014; Soong et al., 2015) care costs with at least €300 million Euro.

In conclusion, rerouting resilience forecasting research from static to dynamic predictors, based on time series of key physiological processes that reflect sub-system equilibrium, is warranted by currently available empirical and mechanistic evidence. Specifically in geriatrics, this would complement advances in frailty research and ultimately the clinical use of resilience measures may improve quality of care and older patients’ wellbeing. To prevent that the “resilience” term, just as “frailty” right now, will soon be used in many undefined different meanings in geriatrics and gerontology, it is best to realize that resilience is already defined in other domains (e.g., pediatrics, public health) in different ways. In this paper, we follow the definition stated by the National Institute of Aging consensus paper (Hadley et al., 2017). As resilience research progresses, it would be best to update this consensus definition from time to time, as, for example, is done in the field of Alzheimer’s disease for the research criteria, in order to prevent misunderstandings, and inadequate research.

## Author Contributions

MO drafted the first version, and MO and RM contributed equally to the revisions. MO and RM contributed equally to subsequent revisions, and MO did the last refinements.

### Conflict of Interest Statement

The authors declare that the research was conducted in the absence of any commercial or financial relationships that could be construed as a potential conflict of interest.

## Footnotes

^1^https://ec.europa.eu/eurostat
